# Spatial analysis of falls in an urban community of Hong Kong

**DOI:** 10.1186/1476-072X-8-14

**Published:** 2009-03-17

**Authors:** Poh C Lai, Chien T Low, Martin Wong, Wing C Wong, Ming H Chan

**Affiliations:** 1 Department of Geography, The University of Hong Kong, Pokfulam Road, Hong Kong; 2 Department of Medicine and Geriatrics, Kwong Wah Hospital, Waterloo Road, Kowloon, Hong Kong; 3 Department of Orthopaedic and Traumatology, Kwong Wah Hospital Waterloo Road, Kowloon, Hong Kong

## Abstract

**Background:**

Falls are an issue of great public health concern. This study focuses on outdoor falls within an urban community in Hong Kong. Urban environmental hazards are often place-specific and dependent upon the built features, landscape characteristics, and habitual activities. Therefore, falls must be examined with respect to local situations.

**Results:**

This paper uses spatial analysis methods to map fall occurrences and examine possible environmental attributes of falls in an urban community of Hong Kong. The Nearest neighbour hierarchical (Nnh) and Standard Deviational Ellipse (SDE) techniques can offer additional insights about the circumstances and environmental factors that contribute to falls. The results affirm the multi-factorial nature of falls at specific locations and for selected groups of the population.

**Conclusion:**

The techniques to detect hot spots of falls yield meaningful results that enable the identification of high risk locations. The combined use of descriptive and spatial analyses can be beneficial to policy makers because different preventive measures can be devised based on the types of environmental risk factors identified. The analyses are also important preludes to establishing research hypotheses for more focused studies.

## Background

Many fall related studies have been conducted since the 1980s [1-6]. These investigations have demonstrated an association between falls and various causal factors. Most of the results suggested that falls were associated with one or more identifiable risk factors and interventions to these risk factors could remarkably reduce the rates of fall [4,7].

Because causes and risk factors of falls are diverse, there is no agreed classification [8]. Most studies examined falls from the epidemiological context [7,9] while others grouped risk factors into intrinsic and extrinsic causes [4,8,10,11]. In general, risk factor studies have been approached in two directions. Some studies examined falls with respect to human induced causes focusing on demographic characteristics (age, sex, history of prior fall), personal conditions (vision, postural instability, chronic illness), medication effects (prescribed medicine, drug), and situational factors (activities engaged at the time of fall, e.g., domestic, recreational, casual). Others looked at falls from the environmental perspectives in relation to indoor (dangerous sets in the home environment) or outdoor (weather, landscape, built structure) hazards. However, distinction between the two approaches is not always clear, as in the case of situational activities which have an environmental setting even though human induced.

The issue of old age, gender and history of falls are described as demographic factors that predispose the elderly to falls. Old age (defined here as 65 or above) has witnessed a greater prevalence and incidence of falls generally associated with physical deficiency or diseases [12,13]. However, further research is needed to assert gender disparity in elderly falls. A number of researchers [12,14-16] claimed that elderly women had a higher risk of sustaining an injurious fall but the findings of Fletcher and Hirdes [17] said otherwise. Research also indicated that individuals with a history of fall tended to suffer from recurrent falls [18] and were three times more likely to incur falls in the future [12].

A mix of medical problems appears as very common causes in elderly falls. Side effects of disease treatment and medication often alter adversely the physical conditions of an individual to cause gait and balance disorders [3,7,13,16,19,20]. Fletcher and Hirdes [17] found impaired gait and balance to associate with not only an increased risk of falls but also recurrent falls. Researchers also established that elderly falls were linked with various medical illnesses or pathological conditions, including diabetes [21], Parkinson's disease [12,17], cardiovascular diseases [22], and cancer [6]. While the contribution of various medications towards falls in the elderly was well founded, Lee et al. [22] contended that some underlying medical illnesses (including eye diseases, heart problems, lower back and leg pain) were actually responsible for falls rather than the medication.

Bath and Morgan [23] suggested that undertaking activities in different locations and circumstances had a relation with the intrinsic risks of a fall. Certain physical activities around a fall (such as light and heavy house work, home repair, lawn work, outdoor-gardening, and caring for another person) were found to increase the risk of falls [9]. Most people are exposed to risks of fall in their home environment [24] but an elderly person situated in improper home surroundings has a greater risk of fall due to slips or trips [5,12,24,25]. On the issue of outdoor environmental risks, Li et al. [26] found that the elderly was more susceptible to falls even though they spent little time outdoors. The majority of fallers reported their underfoot accidents were caused by tripping or slipping on objects or uneven surfaces in these locations.

Todd and Skelton [11] recognized that falls often result from the dynamic interaction of risks in all categories and that the univariate consideration of individual risk factor ignores confounding effects whereby a risk factor may explain another if evaluated in a multivariate manner. Conventionally, a fall patient is assessed from various angles through fall assessment, fall risk assessment and clinical evaluation [10]. Fall assessment takes into account a patient's fall injury and medical problem while fall risk assessment diagnoses individual risk factors that occur simultaneously with the fall. The clinical evaluation, a stage of identification of multiple fall etiologies and risk factors by health professionals, comes after the fall and fall risk assessment have been completed. The contributing risks of fall of an individual will be determined after all evaluations have been completed.

We argue and demonstrate in this paper that an understanding of the spatial variability of falls is an important prerequisite to more elaborate analysis. Descriptive geography helps identify the complex interplay between risk factors to offer insights about the individual-level and contextual factors contributing to falls at specific locations. Such an analysis helps to delimit zones of high risks and target possible causes to enhance the effectiveness of injury prevention programs.

## Methods

### Study Area

**Figure 1:**
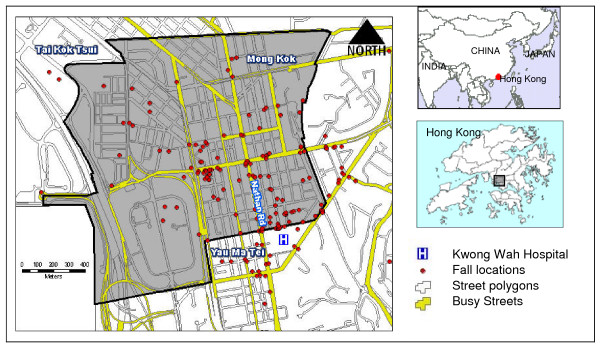
**Fall locations in Mong Kok, Hong Kong, 2006–07**.

The study area of this research is Mong Kok, an urban community covering an area approximately of 268.2 hectares in the Yau Tsim Mong District (Figure 1). Mong Kok is one of the most crowded communities in Hong Kong and had a population density of 40,136 persons per square kilometer in 2006 [27]. With its substantial commercial and retail activities, the peak time pedestrian flow of Mong Kok in 2007 reached 25,000 persons per hour [28]. The overwhelmingly crowded streets and sidewalks mean inadequate space for pedestrian movements and an increased risk of fall.

### Data Collection

Our data collection exercise involved patients attending the Kwong Wah Hospital (KWH) because there is no available official statistics on either the number or cause of falls within the population. KWH is a public hospital serving residents in neighboring districts of Yaumatei, Mong Kok, and Tai Kok Tsui (Figure 1). Nurses at the triage station sought consent of patients with fall-related injuries to participate in the study. The survey was conducted over the telephone to include data on the location (open market, refuse collection points, bus stops, traffic lights) and circumstances (push by others, short signal timing for pedestrian crossings, insufficient lighting, wet surface) of the fall incidents, demographic characteristics of the fallers (age, gender, history of fall), personal traits (gait and balance, visual condition), fall injury, past medical history and long term use of medication [29]. A typical telephone interview session took 5 minutes and the call was made as soon as a case was reported given that some subjects were unable to recall details of an incident that happened a week ago.

The data were gathered in two phases: the pilot phase (July to September 2006) and the second phase (January to December 2007). Interviews in the pilot phase of data collection were conducted face-to-face by a physician at the KWH to refine questions and interview protocols. Subsequent interviews for phase 2 of the study were conducted by a research assistant over the telephone. The collected data were classified into three major categories: outdoor (57.6%), indoor (13.3%) and excluded (29.1%) cases. Reasons for exclusions include no response or refusal to be interviewed, missing or incorrect telephone number, incomplete survey and/or descriptions about the location of fall, fall occurring outside of Hong Kong, or subject has deceased. Only outdoor cases (comprising of 123 and 158 useable records respectively from the pilot and second phases) were used in this study.

All human-related research studies in Hong Kong must undergo a review of human research ethics. This study was exempted from the review of confidentiality because the interview was conducted with consent and the location of fall was in the outdoor. Personal data about demographic characteristics and relevant illnesses were collected from the subjects without revealing the identity of individuals.

### Methods of Analysis

A fall incident can be a multi-factorial occurrence and may involve diverse circumstances and environmental hazards. A descriptive analysis of outdoor falls is essential to offer a general overview of characteristics of the sample population and the situational setting. This is followed by a series of spatial analyses which aim to identify those factors which converge in space to produce falls. Understanding the spatial characteristics of falls augments our ability to draw insights about the locations and causes of falls [30].

A map of points is a map of event locations which reveals the spatial arrangement of the incidents [31]. Fall locations are represented as point features and plotted on a base map with street networks (Figure 1). The procedure required the subjects to offer a nominal address for the location of the fall such as the building name, road name, major road intersections, bus stations, etc. Coordinates of these addresses were then geo-referenced [32] from the Centamap which is a free web map service for Hong Kong launched in 1999. The street networks are very important as most of the fall incidents were found along major roads and streets and at the junctions.

Mapping fall occurrences at the micro-level location, i.e. as points, provides a good visualization aid for small area studies to extract possible environmental causes regarding the distribution of the set of locations before proceeding to more complicated analyses. We can observe a tendency for some cases to occur more closely in space through spatial clustering of fall occurrences. These clusters are considered hot spots of adverse conditions or locations with environmental attributes conducive to falls.

The study made use of the Nearest neighbour hierarchical (Nnh) clustering method, available from the CrimeStat software [33], to delimit standard deviational ellipses (SDE) of hot spots. The SDE is a summarizing tool and an areal graphic representation used to reveal the dispersion and orientation of points around the mean center of a cluster [34,35]. The Nnh is a constant-distance clustering routine that groups points on the basis of their spatial proximity [33]. This study made use of the agglomerative approach [36] to define clusters by grouping two closest incident locations into a cluster. The procedure will halt when the distance between centers of the clusters is greater than some defined threshold distance and minimum points per cluster. The hierarchical approach is adopted because it minimizes dissimilarity (measured in terms of spatial separation between closest pair of representative points) associated with each grouping. Different cluster sizes of 3 to 5 and search radii of 50 m and 100 m were examined by Monte Carlo simulation to explore the sensitivity and statistical significance of these parameters.

The Nnh and SDE techniques, based on criteria parameters defined above, were employed to examine the spatial spread of falls by different attributes. The spatial distribution of falls was studied from the perspectives of gender, age, footwear, floor condition, time of fall to reveal spatial similarities and differences. The multi-factorial nature of falls can be ascertained and the various causes identified to help target preventive measures at specific locations.

## Results

### Descriptive Analysis

Table 1 summarizes the frequencies of outdoor falls with selected characteristics by gender and age group. Discounting indoor falls and excluded cases, 281 locations of outdoor falls identified in both phases of the survey were combined for further analyses. The largest group in the study population, accounting for 72%, were those aged 65 years and older among which 67% were female. 98.6% of the outdoor falls were precipitated by one or more (not mutually exclusive) environmental causes such as uneven and wet or slippery floors. Among those who fell outdoor, 78% tripped over an uneven surface (curb, loose brick, obstacle, uneven pavement, stair and step) and 42% slipped on a wet or slippery surface. 30% of the reported cases fell on a surface with both conditions (i.e. uneven and wet or slippery). Only 1.4% was not sure about the causes of their falls.

**Table 1:** Frequency of outdoor falls with selected characteristics by gender and age groups

**Characteristic**	**Frequency**									**(%)**
		
	**Female**				**Male**				**TOTAL**	
		
	**20–44**	**45–64**	**> 64**	**Total**	**20–44**	**45–64**	**> 64**	**Total**		
		
	**(n = 14)**	**(n = 48)**	**(n = 146)**	**(n = 208)**	**(n = 6)**	**(n = 10)**	**(n = 57)**	**(n = 73)**	**(N = 281)**	
**Environmental Cause**										

Uneven floor	11	41	113	**165**	3	4	46	**53**	**218**	**78**

*curb*	*1*	*6*	*10*	***17***	*0*	*1*	*6*	*7*	*24*	*9*

*Loose bricks*	*1*	*2*	*6*	***9***	*0*	*0*	*4*	*4*	*13*	*5*

*Obstacle*	*2*	*4*	*16*	***22***	*0*	*0*	*5*	*5*	*27*	*10*

*Steps*	*1*	*2*	*11*	***14***	*0*	*0*	*6*	*6*	*20*	*7*

*Stairs*	*3*	*8*	*2*	***13***	*0*	*1*	*2*	*3*	*16*	*6*

*Uneven pavement*	*3*	*19*	*68*	***90***	*3*	*2*	*23*	***28***	***118***	***42***

Slippery/Wet Floor	6	17	66	**89**	3	2	23	**28**	**117**	**42**

Any of above	1	10	54	**65**	1	0	17	**18**	**83**	**30**

Uncertain	0	2	1	**3**	0	1	0	**1**	**4**	**1**

**Activity during fall**										

Walking	10	39	109	**158**	4	6	41	**51**	**209**	**74**

Rushing	4	8	31	**43**	2	3	10	**15**	**58**	**21**

Morning Exercise	0	0	2	**2**	0	0	4	**4**	**6**	**2**

Alighting from bus	0	1	4	**5**	0	1	2	**3**	**8**	**3**

**Specific Complaints**										

Insufficient Light	1	8	7	**16**	1	1	6	**8**	**24**	**9**

Avoid car hitting	0	0	2	**2**	0	0	0	**0**	**2**	**1**

Short signal timing for pedestrian crossings	0	0	2	**2**	0	0	2	**2**	**4**	**1**

Pushed by pedestrian	1	1	13	**15**	2	0	3	**5**	**20**	**7**

Hit by wheelbarrow	0	1	0	**1**	0	1	0	**1**	**2**	**1**

**Time of fall**										

Midnight to 6 am	0	2	3	**5**	0	0	3	**3**	**8**	**3**

6 am to Noon	6	21	86	**113**	2	5	32	**39**	**152**	**54**

Noon to 6 pm	5	19	50	**74**	2	3	18	**23**	**97**	**34**

6 pm to Midnight	3	6	7	**16**	2	2	4	**8**	**24**	**9**

										

Weekend	2	11	55	**68**	2	1	18	**21**	**89**	**32**

Weekday	12	37	91	**140**	4	9	39	**52**	**192**	**68**

										

Raining	4	7	32	**43**	2	1	15	**18**	**61**	**22**

Clear	10	41	114	**165**	4	9	42	**55**	**220**	**78**

**Footwear**										

Sandal	0	15	80	**95**	2	5	25	**32**	**127**	**45**

Sport Shoes	2	6	13	**21**	2	2	8	**12**	**33**	**12**

Proper Shoes	5	21	53	**79**	2	2	24	**28**	**107**	**38**

High Heels	7	5	0	**12**	0	0	0	**0**	**12**	**4**

others	0	1	0	**1**	0	1	0	**1**	**2**	**1**

**Injury**										

Minor	14	38	86	**138**	5	7	40	**52**	**190**	**68**

Serious	0	10	59	**69**	0	3	16	**19**	**88**	**31**

*not sure	0	0	1	**1**	1	0	1	**2**	**3**	**1**

**Hospitalization**										

Yes	0	5	31	**36**	0	1	10	**11**	**47**	**17**

No	14	43	115	**172**	6	9	47	**62**	**234**	**83**

**Use of assistive device**										

Yes	0	3	55	**58**	0	0	23	**23**	**81**	**29**

No	14	45	91	**150**	6	10	34	**50**	**200**	**71**

**Gait**										

Stable	12	38	65	**115**	6	7	21	**34**	**149**	**53**

Unstable	2	10	81	**93**	0	3	36	**39**	**132**	**47**

**Vision**										

Clear	14	43	57	**114**	6	8	22	**36**	**150**	**53**

Unclear	0	5	89	**94**	0	2	35	**37**	**131**	**47**

**Fall in most recent 5 years**										

Yes	2	6	80	**88**	0	5	37	**42**	**130**	**46**

No	12	42	64	**120**	6	5	20	**31**	**151**	**54**

All groups considered, the highest percentage of outdoor falls at 74% occurred when a subject was walking. The result agrees with the findings of Li et al. (2006) that walking was the most common physical activity across all age groups. Other fall-related physical activities included rushing (21%), alighting from buses (3%) and doing morning exercises (2%). Non-activity related complaints associated with falls included insufficient light (9%), pushed by others (7%), avoid hitting by vehicles (1%), short signal timing for pedestrian crossings (1%) and hit by a wheelbarrow (1%).

Most of the outdoor falls occurred between 6 am and 12 noon, followed by 12 noon to 6 pm. These two time slots accounted for almost 89% of all outdoor falls in the study. The rest happened between 6 p.m. to 6 a.m. 32% of falls were recorded during the two-day weekends while 68% were recorded during the weekdays. The majority of fall incidents (78%) occurred on a clear day. Only 22% of the subjects fell on a rainy day.

The majority or 45% of fallers wore sandals while a 4% minority had high heels at the time of fall. 38% and 12% respectively wore proper shoes and sport shoes with the remaining 2% of other shoes not of the above categories. 47% of fallers claimed that they had an unbalance gait but only 29% were using an assistive device such as an umbrella or a stick. Also, 47% of fallers claimed that they had unclear vision.

Nearly all fallers in this study sustained an injury. More than a half of which or 68% of fallers suffered minor injuries such as abrasions, sprains and lacerations. 31% of falls resulted in major injuries such as fractures, dislocations, head injuries and haemorrhage. 1% walked away and left the hospital after registration without any diagnosis. For those diagnosed by a doctor at the KWH, 17% were requested to stay in the ward for further treatments and observations. Among the fallers, 46% experienced a fall within the last five years prior to this study.

### Spatial Analysis

The descriptive analysis above provides an aspatial contextual summary of the sample population. Certain environmental (uneven and wet or slippery floors), activity-based (walking or rushing) and temporal (different time slots) causes reported significantly higher percentages to warrant attention. However, the above findings fall short of indicating the whereabouts of these environmental hazards and locations of specific characteristics deemed more susceptible to falls.

The Nnh clustering method identifies a hot spot by grouping points of close proximity to each other. An advantage of the Nnh method is that hot spots can be detected at different spatial scales. Although a hot spot as delimited using the SDE may imply an extent small in area coverage, there is no rule of thumb for its size. In this regard, the clustering method was experimented for several runs to get an acceptable combination of criteria (i.e. the minimum number of points in a cluster and the threshold distance).

**Figure 2:**
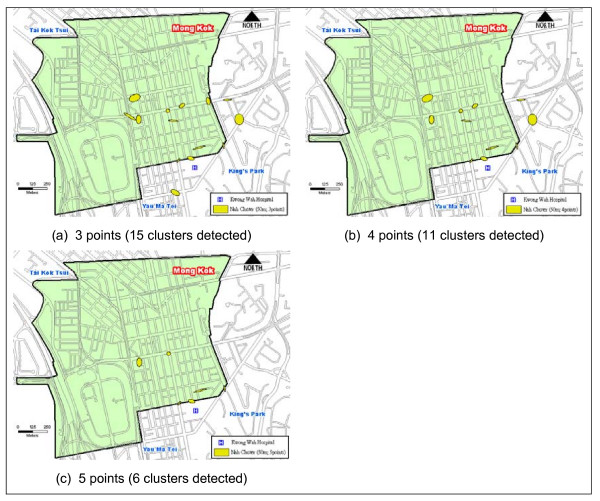
**A comparison of Nnh clusters using 50 meters as the threshold distance and varying minimum points per clusters**.

Figure 2a, Figure 2b, and Figure 2c illustrate the dissimilarity of Nnh clusters drawn on a threshold distance of 50 meters by using different minimum number of points per cluster. Most of the clusters designated with SDEs are compact in shape and approximately of sizes 400–5300 square meters. The ellipses are small and roughly of the size of a junction. The size of the hot spots dictates the extent of the search areas to target site investigation.

**Figure 3:**
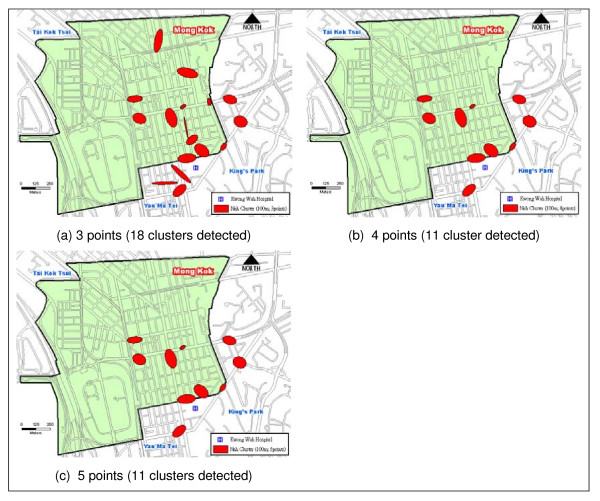
**A comparison of Nnh clusters using 100 meters as the threshold distance and varying minimum points per clusters**.

Figure 3a, Figure 3b, and Figure 3c, however, show another set of Nnh clusters based on a threshold distance of 100 meters. By increasing the threshold distance one-fold larger, the maximum coverage of the clusters increases to 12,300 square meters. As a result, some of the hot spots shown in Figure 1a (e.g. clusters near the KWH) begin to encroach upon each other and become a bigger hot spot. An increase in the threshold distance has resulted in hot spots of a larger areal coverage encompassing more than one junction, which can lead to a loss in focus (i.e., the exact location of problematic areas within a hot spot may be obscured).

The Monte Carlo simulation was executed 1000 times to test the significance level of the search threshold distance and minimum number of points per cluster (Table 2). At the 95% confidence interval on a two-tailed test (i.e. at the 2.5^th ^and 97.5^th ^percentile), the simulation of 50 meters and 3 points indicates that the result expects 14 clusters to be "real" and one cluster (i.e. 15 observed clusters minus 14 significant clusters) to occur just on the basis of randomness. In a real cluster, fall cases occur in temporal and spatial proximity at a higher frequency than by chance. The simulation did not show which clusters were real but it provided a statistical basis to evaluate the number of clusters that did not occur purely by chance. Table 2 also shows that the minimum number of points per cluster must not be too few. The combination of 50 meters and a minimum of 4 or more points, in this case, yields a significantly stable number of 11 real clusters. The same observation applies for those using 100 meter as the threshold distance although all but one of the clusters is real.

**Table 2:** Results of Monte Carlo simulations on the Nnh clustering method

Criteria			
		
Threshold distance	Minimum points per cluster	Number of Observed Clusters	Numbers of clusters identified at the 95% confidence interval
***50 m***	3 points	15	14
	
	***4 points***	***11***	***11***
	
	5 points	6	6

100 m	3 points	18	15
	
	4 points	11	10
	
	5 points	11	10

**Figure 4:**
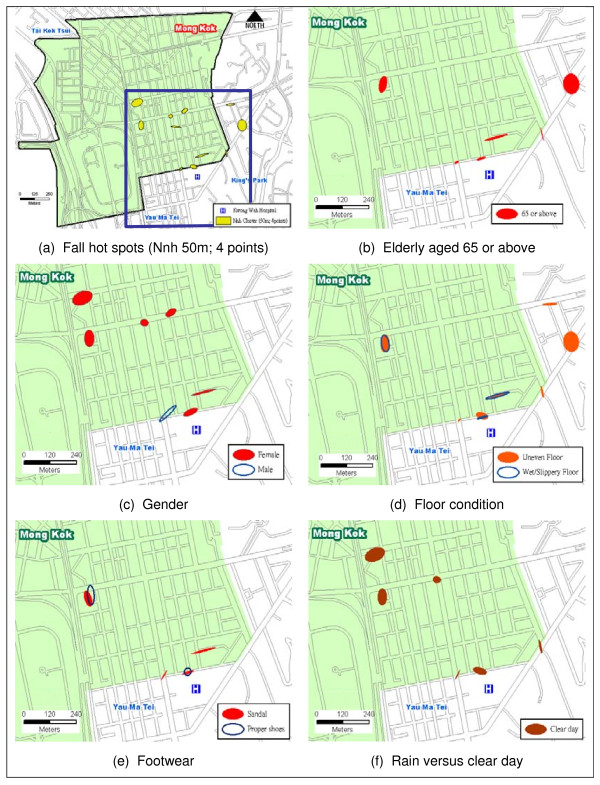
**Spatial spread of falls by non-spatial characteristics (I)**.

The Nnh cluster analysis, using a threshold distance of 50 meters and 4 minimum points per cluster, can be taken to the next level to examine the spatial spread of falls by non-spatial characteristics (e.g. age, gender, floor condition, footwear, etc.). Figure 4b shows that six of the eleven clusters involved elderly falls. Figure 4c shows that more females than males were involved in the fall hot spots while Figure 4d and Figure 4e show the multi-factorial nature of falls involving a combination of uneven, wet/slippery floors, and improper footwear. Figure 4f indicates that the fall hot spots were not attributed to rainy days which could not have been the cause of wet/slippery floors identified in Figure 4d.

**Figure 5:**
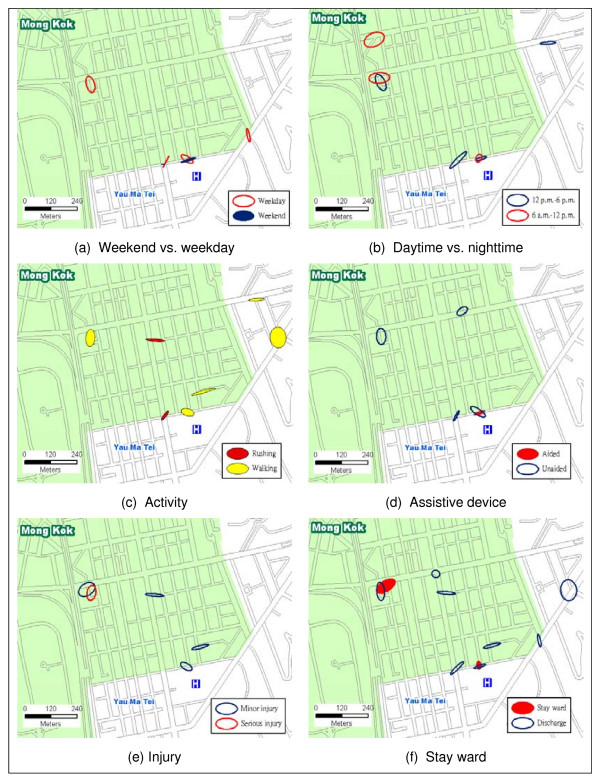
**Spatial spread of falls by non-spatial characteristics (II)**.

Similar to Figure 4, The Nnh clusters and SDEs in Figure 5 reveal environmental characteristics of the fall hot spots from the spatial distributional patterns. Figure 5a and Figure 5b collectively show that fall hot spots had a spatial-temporal dimension. Figure 5a shows that four of the six hot spots involving the elderly occurred on weekdays. Figure 5b however highlights daytime versus nighttime hot spots of falls. Interestingly, Figure 5c and Figure 5d when compared against Figure 4b unveils the fact that elderly falls happened mostly while walking without assistive device. While most individuals ended up with minor injury without the need to stay in the ward as illustrated in Figure 5e and Figure 5f, one of the hot spots involving the elderly actually did register an unusually high incidence of serious injury.

**Figure 6:**
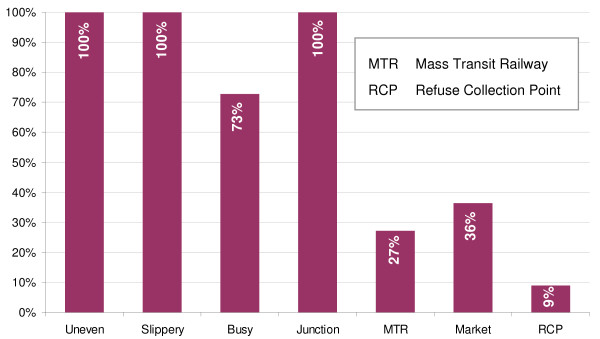
**Multiple reasons of falls at the 11 identified fall hot spots**.

The multi-factorial nature of the 11 fall hot spots is summarized in Figure 6. Almost all of the fall hot spots occurred in the vicinity of busy (73%) junctions characterized by uneven and slippery surfaces. These locations included 3 station entrances/exits to the Mass Transit Railway (MTR), 4 outdoor markets, 1 refuse collection point, and 3 busy street junctions. Interestingly, fall locations at the not-so-busy junctions were found in the outdoor markets.

The descriptive and spatial analyses above add insights into the sampled group of fallers and offer essential groundwork studies before formalizing research hypotheses for further examination. Notwithstanding the somewhat qualitative nature of the analysis, an understanding of the environmental and situational characteristics of hot spots does help identify and pinpoint locations of specific environmental hazards for selected groups of the population.

## Discussions and Conclusion

Places or locations of outdoor falls are fundamental components to enable a better understanding of the geographic nature of outdoor-related fall injuries that often vary in a community. Understanding geographic characteristics of fall injury in a study area is the first step to target the right place and population at risks. At the elementary level, mapping by way of point symbols offers the simplest and detailed presentation of discrete locations of falls. Point representation of fall location is more effective than areal count data in a small community (e.g. Mong Kok) because it conveys much more information in a large scale study area. The spatial characteristics of an outdoor fall are also more revealing through visualization of a point map. Additional knowledge about the locational characteristics of falls widens the horizons and scopes of such a study.

Visualization is an essential part in spatial epidemiology to discover new patterns that otherwise would have remained invisible in a conventional database table [37]. Given that a fall incident often has multiple factors interrelated to each other, the interplay between these factors can be examined from the spatial perspective and in an organized way. This study demonstrates the use of Nnh and SDE techniques to detect hot spots of falls and to inspect possible environmental causes for falls at these locations. Explanations about the clustering of outdoor falls are independent and may differ over space and time. It is important to consider specific characteristics of the built environment to explain the clustering phenomenon in a particular area. In this regard, identifying hot spots makes targeting geographically specific prevention programs feasible.

The collected sample shows that outdoor falls among the elderly was as common as indoor-related fall. The frequency of fall incidents among the elderly is higher than any other age groups and six of the eleven hot spots of falls had cases involving the elderly. Uneven and wet or slippery floors were two major reasons for the fall incidence. Hazards associated with walking that are known and confirmed in this study to cause trips or slips include street curbs, loose bricks, obstacles, steps, stairs, uneven and slippery pavements, and damp or wet surfaces.

Environment-related hazards are often region-specific and dependent upon different built features or landscape characteristics. The approach employed in this study yields meaningful results and the knowledge accumulated from this research also provides some guidelines for the design of a walker-friendly environment in Hong Kong. For example, it is a known fact that people slip and fall on wet and slippery grounds. Our spatial analysis helps to pinpoint the exact locations of wet/slippery or uneven ground needing attention. This approach is particularly helpful for locating problem spots within an outdoor market in Hong Kong which may stretch across several street blocks. A less-slippery material such as permeable concrete pavements should be used for these problem spots and regular resurfacing may be warranted.

**Figure 7:**
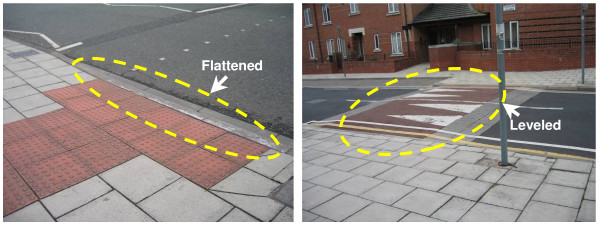
**Examples of flattened and leveled street curbing**.

In reference to fall hot spots in the vicinity of MTR stations, ramps instead of short steps are preferred because a sudden or abrupt change in elevation can cause trips. Fall hot spots at the busy street junctions coincide with pedestrian crossings. Here, street curbing should be flattened or leveled (Figure 7) to reduce risks of tripping and to enhance walkability. The signal timing should be tuned to allow more time for pedestrian clearance. Furthermore, the single refuse collection point identified through this approach should perhaps be relocated away from a busy junction next to an outdoor market.

Acquiring good quality data is a key factor in health-related research to earn reasonably reliable results. Data for this research were collected in collaboration with doctors and nurses of a local hospital. It is arguable that the data may not be representative of the real pattern of falls in the study area because injuries either treated in other hospitals or by other means (e.g. traditional Chinese massage therapy) or not treated will not be included. Furthermore, the data screening program of this research was conducted on a voluntary basis by patients who gave their consents for the follow-up survey. Nonetheless, the study demonstrates the value of the spatial analysis approach in enlightening environmental concerns about falls.

Our study offers empirical evidence about the complex interplay between multiple causes of falls and the environmental setting. The approach is one of spatial ecology aimed at studying the distribution and concentration of fall incidents over time. It presents not only a mechanistic understanding of the temporal and spatial variation of falls but also possible existence of the synchronous dynamics. The multi-factorial nature of falls has been studied typically by statistical and correlation analyses of time-series data. There are limitations to what inferences can be made about mechanisms inducing falls from population time-series data alone. The incorporation of the spatial aspect enables concentration of fall events at different locations to be discriminated between environmental, temporal, and human variations. The concept of spatial clustering based on distance between event locations is effective in identifying driving variables (whether environmental, temporal, or human) operating at a local scale because the tendency to cluster is not expected if different locations are being influenced by the same.

## Abbreviations

KWH: Kwong Wah Hospital; MTR: Mass Transit Railway; Nnh: Nearest neighbour hierarchical (clustering method); SDE: Standard deviational ellipse.

## Competing interests

The authors declare that they have no competing interests.

## Authors' contributions

PCL supervised the study, participated in the research design and analysis, and drafted the manuscript. CTL carried out the spatial analysis. MW and WCW participated in the design of the data collection procedures. MHC participated in its design and coordination of the study. All authors read and approved the final manuscript.
